# A Review of Robotic Surgery in Colorectal Surgery

**DOI:** 10.7759/cureus.37337

**Published:** 2023-04-09

**Authors:** Kapilraj Ravendran, Emmanuel Abiola, Kowthaman Balagumar, Ahsan Z Raja, Mohammed Flaih, Sonny P Vaja, Alhad O Muhidin, Nikolaos Madouros

**Affiliations:** 1 Medicine, Gradscape, London, GBR; 2 Surgery Working Group, Society of Junior Doctors, Athens, GRC

**Keywords:** colorectal surgery, davinci, anterior resection, colorectal cancer, robotic

## Abstract

Colorectal surgery is a treatment for colorectal lesions. Technological advancements have given the rise to robotic colorectal surgery, a procedure that limits excessive blood loss via 3D pin-point precision capabilities during surgeries. The aim of this study is to review robotic surgery in colorectal treatment procedures in order to dictate its ultimate merits. This is a literature review utilising PubMed and Google Scholar; it only includes case studies and case reviews related to robotic colorectal surgeries. Literature reviews are excluded. We incorporated abstracts from all articles and full publications were examined to compare the benefits of robotic surgery in colorectal treatments. The number of articles reviewed was 41 literature spanning from 2003 to 2022. We found that robotic surgeries yielded finer marginal resections, greater amounts of lymph node resections and earlier recovery of bowel functions. The patients also spent less time in hospital after surgery. The obstacles on the other hand are it costs longer operative hours and further training, which is expensive. Studies show robotic approach is a choice for treating rectal cancer. However further studies would be needed to conclude the best approach. This is especially true with patients treated for anterior colorectal resections. Based on the evidence it's safe to say that the upsides outweigh the downsides, but advancements and further research in robotic colorectal surgeries are still necessary to reduce operative hours and cost. Surgical societies should also take the initiative and set up effective training programmes for colorectal robotic surgeries, as trained physicians result in better treatment outcomes.

## Introduction and background

Colorectal surgery includes procedures such as polypectomy, hemicolectomy, partial colectomy, segmental resection and total colectomy which help treat colorectal diseases such as polyps, cancers, diverticulitis, bowel blockage, etc. [1]. These procedures are done by either open, laparoscopic or robotic procedures [1]. In the last two decades, there has been a trend to opt for minimally invasive procedures [1]. Despite initial doubts, it has grown since first being described in 1991 and has now become the standardized care in western countries for benign and malignant colorectal diseases [2]. There are limitations to the procedures though [2], the main ones being that laparoscopic colorectal surgery is two-dimensional (2D), the assistant-dependent camera is unstable, poor ergonomics, straight fine tip instruments and effects of tremor [2].

To overcome these obstacles, robotic surgical systems were designed to try to overcome these limitations [2]. Several robotic devices have been developed [2]. The da Vinci system (Intuitive Surgical, Sunnyvale, CA, USA) is considered to be the first system approved by the FDA in 2000 [2]. The first report from Weber and colleagues who performed a right hemicolectomy and sigmoid colectomy for benign disease was in 2002 [2]. The first robotic colectomies were performed in 2001 and has an increasing incidence ever since [3]. It is being used in not only benign cases but in malignancies as well [3]. The operating surgeon controls the camera using a foot peddle and also gives the operating surgeon a highly stable surgical view [3].

It is believed that the robotic approach can potentially resolve the limitations of elective standard laparoscopic rectal surgery [4]. Laparoscopic and robotic surgery are being distinguished by new technologies, as minimally invasive surgery develops [5]. Modern surgery may significantly benefit from the incorporation of robotic technology [5]. The device converts the surgeon's normal hand movements into exact movements of the laparoscopic instruments within the patient [5]. The robotic approach is said to have an equivalent efficacy and safety to the laparoscopic approach, broadening its application [6]. Moreover robot's prospective technological advantages over traditional laparoscopy have sparked interest in extending its use to colorectal operations [6]. 

The aim of this study was to review robotic surgery in colorectal procedures in order to outline its value over other minimally invasive techniques. The study is an analytic review of robotic cases in colorectal surgery, with emphasis on indications of using robotic systems. As far as we are aware, no recent studies have been made on this matter.

## Review

Methodology

**Figure 1:**
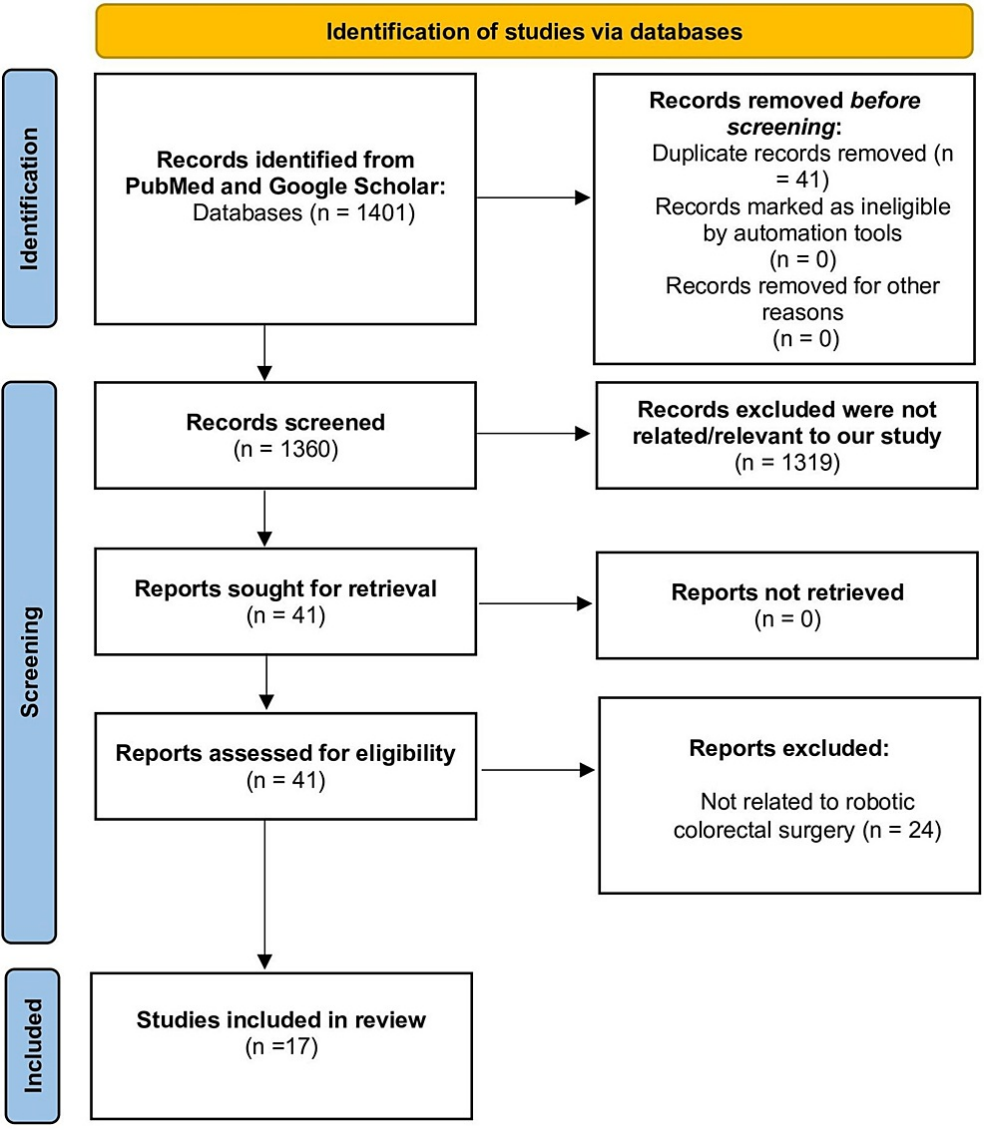
PRISMA flow diagram demonstrating the literature selection strategy. PRISMA = Preferred Reporting Items for Systematic Reviews and Meta-Analyses

This is a literature review where articles were searched and reviewed using PubMed and Google Scholar. The keywords used were “robotic assisted surgery” combined with Medical Subject Headings (MeSH) “colorectal surgery”. Abstracts from all articles were obtained and full texts were examined and considered to compare the benefits of robotic surgery in colorectal surgery. Eight authors screened titles and abstracts in English for relevant studies from 2003 to 2022. Our inclusion data was related to robotics used in colorectal surgery, which were abstracted from each study and included in our review. A total of 1401 articles were identified. Forty-one duplicate articles between the different databases were removed. After screening, by going through the titles, abstract and full article, only 41 studies were left. Twenty-four more studies were excluded as they focused mainly on laparoscopic and other surgical procedures, and not robotic surgery. Our study only included case reports and case reviews. All literature reviews were removed from our study. Seventeen relevant studies were included after thorough screening. The literature search was conducted and the Preferred Reporting Items for Systematic Reviews and Meta-Analyses (PRISMA) flow chart provided showcases this (Figure 1) [7].

Results

The initial search yielded 41 relevant publications, following the exclusion of non-primary sources from the database search and the deletion of duplicates. After screening titles and abstracts (n=41) and excluding 24 records on the grounds of irrelevance to the topic, the full texts of 17 articles were assessed which focused on robotic versus laparoscopic approach and included six cohorts for right hemicolectomy, seven cohorts for colectomies and five cohorts for left hemicolectomy and anterior resection.

Right Hemicolectomy

For right hemicolectomy, there were 436 patients who underwent robotic surgery [8-13]. These studies showed robotic surgery to have similar outcomes to laparoscopic surgery [8-13]. Robotic surgery had longer operative times, but had better resection margins and more lymph node harvested as well as less post operative hospital stay [8-13]. Studies found that robotic colorectal surgery for right hemicolectomy patients had similar outcomes to laparoscopic surgery [8-11]. Table 1 summarises our findings for right hemicolectomy.

**Table 1:** Right Hemicolectomy Mins = Minutes; ml = millilitre

Source	No of Patients	Operative Time	Blood Loss	Post Op flatus and bowel opening	Conversion rate	Anastomosis leak	Wound Infection	Other Complications	Number of harvested lymph nodes	Hospital stay
Conor P Delaney et al [8]	Robotic – Two patients Laparoscopic – Two patients	Robotic: - 267 – 274 mins VS Laparoscopic: - 116 – 160 mins	Robotic:- 100ml VS Laparoscopic: 100-200ml	-	-	-	-	Robotic: - one atelectasis VS Laparoscopic - Zero	-	Robotic: - 2-5 days VS Laparoscopic: 2-3 days
Nima Ahmadi et al [9]	Robotic - 59 patients Laparoscopic - 42 patients	Perioperative Robotic: - 110min VS Laparoscopic: - 97 mins	Robotic 53.62 ± 34.02 VS Laparoscopic 63.57 ± 35.21	Median Flatus One day for robotic VS three days for laparoscopic Median Bowel opening Two days for robotic VS Four days for laparoscopic	-	Robotic – zero VS Laparoscopic - zero	-	Robotic less likely to have ileus than laparoscopic	18 lymph nodes for robotic VS 14 for laparoscopic	Median Robotic: - Three days VS Laparoscopic: - Five days
M Benjamin Hopkins et al [10]	79 patients Robotic - 17 patients Laparoscopic – 62 patients	Median Room Time Robotic 285 mins VS Laparoscopic: - 170 minutes Median Procedure Time Robotic: - 203 minutes VS Laparoscopic: - 118 minutes	-	-	Robotic - Zero VS Laparoscopic - Five	Robotoc - One VS Laparoscopic - Zero	-	Robotic (two ileus) VS Laparoscopic (two ileus) Readmission: - Robotic (one) VS Laparoscopic (Three)		Robotic - 2,54 days VS Laparoscopic - 3.27 days
L Solaini et al. [11]	Robotic - 305 patients laparoscopic – 84 patients	Median operative time Robotic - 250 mins VS Laparoscopic - 160 mins	Robotic - 50ml VS Laparoscopic - 50ml	Median Flatus Robotic - Three days VS Laparoscopic - Two days	Robotic - Three VS Laparoscopic - Zero	Robotic - Eight (2.6%) VS Laparoscopic - Three (3.6%)	Robotic - 6.8% VS Laparoscopic - 7.1%	Robotic - 23.3% VS Laparoscopic - 25% f	Median lymph nodes Robotic - 22 VS Laparoscopic - 19	Median Robotic- Seven days VS Laparoscopic - Eight days
Valentina Ferri et al [12]	Robotic - 35 patients Laparoscopic - 35 patients	Mean operative Robotic -243 mins VS Laparoscopic - 179 mins	-	Robotic - 2.7 days VS Laparoscopic - 3 days	Robotic 28 VS Laparoscopic- 29	-	- - -	Robotic - 8 patients VS Laparoscopic - 9 patients	-	Robotic - 8.3 days VS Laparoscopic - 8.7 days
Mario Guierrieri et al [13]	Robotic – 18 patients Laparoscopic 3D – 11 patients	Overall Time Robotic - 173 (156-189) mins VS Laparoscopic - 145 (130-155) mins Surgical Time Robotic - 130 (106-139) mins VS Laparoscopic - 100 (95-120) mins	-	Robotic – One day VS Laparoscopic - Two days	Robotic – One VS Laparoscopic - Two1		-	Robotic – Three VS Laparoscopic – Two		Robotic - Five days VS Laparoscopic – Five days

Left-Sided or Rectal (High and Low Anterior) Colorectal Resections

Three hundred forty patients had robotic surgery for either left hemicolectomy or lower anterior resection [13-17]. Robotic surgery had longer operative times but had earlier postoperative flatus and bowel openings as well as less conversion rates [13-17]. Robotic surgery also had less anastomotic leaks, less complications and better resection margins [13-17]. Patients who underwent robotic surgery also had less hospital stay [13-17]. Hettiarachchi et al. concluded that robotic surgery for anterior resection significantly reduced postoperative hospital stay and can also reduce the need for converting to open surgery [16]. The surgeon typically becomes more proficient in laparoscopic low anterior resection for rectal cancer after 50 cases [18]. Gass et al. concluded that robotic-assisted, left-sided colectomies were safe and feasible compared to laparoscopic resections [17]. Compared to laparoscopic resections, the analysed robotic-assisted resections have longer operative times but less conversion rates [17]. Laparoscopic had an 8% conversion rate, while robotic had zero [19]. Furthermore, the robotic approach was associated with lower conversion rates (1-7.3% compared with 3-22%) [19]. Table 2 summarises our findings for left hemicolectomy and anterior resection.

**Table 2:** Left-Sided or Rectal (High and Low Anterior) Colorectal Resections R-TME = robotic total mesorectal excision; L-TME = laparoscopic total mesorectal excision; mins = minutes; R-LAR = robotic left anterior resection; L-LAR = laparoscopic left anterior resection

Source	No of Patients	Operative Time	Blood Loss	Post Op flatus and bowel opening	Conversion rate	Anastomosis leak	Wound Infection	Any dysfunction	Other Complications	Number of harvested lymph nodes	Hospital stay
P P Bianchi et al [14]	50 patients Robotic (R-TME) - 25 patients Laparoscopic (L-TME) – 25 patients	R-TME – 240 mins VS L-TME – 237 mins	-	Median bowel opening times R-TME – Two days VS L-TME – Three days	R-TME - Zero VS L-TME - One	R-TME - One VS L-TME - Two	R-TME - One VS L-TME - Two	-	R-TME - 16% VS L-TME - 24%	Median lymph nodes R-TME - 18 VS L-TME - 17 Circumferential R-TME – Zero VS L-TME - one	R-TME – 6.5 days L-TME – six days
Seung Hyuk Baik et al [15]	113 patients Robotic ( R-LAR) – 56 patients Laparoscopic (L – LAR)- 57 patients	Robotic - 190.1 ± 45.0 mins VS Laparoscopic - 191.1 ± 65.3 mins	-	Mean – Robotic - 1.9 days VS Laparoscopic - 2.1 days	Zero for R -LAR VS 10.5% for L-LAR	One for R-LAR VS Four for L-LAR	-	-	10.7% for R-LAR VS 19.3% for L-LAR	Mean lymph nodes Robotic - 18.4 VS Laparoscopic - 18.7 Mean proximal resection margin Robotic - 10.9±4.0 cm VS Laparoscopic -10.8±4.3 cm Mean distal resection margin Robotic -- 4.0±1.6cm VS Laparoscopic - 3.6±1.7 cm	Robotic - 5.7 days Laparoscopic - 7.6 Days
Hettiarachchi, T.S et al [16]	184 Patients Robotic -74 patients Laparoscopic – 110 patients	Median Robotic - 308 mins VS Laparoscopic - 326mins			Robotic - 0% VS Laparoscopic - 16%	Robotic (three) - 4% VS Laparoscopic (six) - 5.5%			Robotic -12,2% VS Laparoscopic - 20%	-	Robotic - three days Laparoscopic - five days
John-Markus Gass et al [17]	Robotic – 179 patients Laparoscopic – 504 patients	Robotic - 300 mins- VS Laparoscopic -- 210 mins	- -		Robotic - 1.7% VS Laparoscopic - 6.1%	Robotic - Two (1.1%) VS Laparoscopic - Eight (1.6%)			Intraoperative complication Robotic - 0.6% VS Laparoscopic - 2.0% Post operative complications Robotic - 3.9% VS Laparoscopic - 6.3%		Robotic - nine days Laparoscopic - nine days
Mario Guierrieri et al [13]	Robotic – six patients Laparoscopic 12 patients-	Median operative time Robotic - 100mins VS Laparoscopic - 167mins	-- -	Robotic – Two days VS Laparoscopic – Two days	Robotic – One VS Laparoscopic – One	-			Robotic – One VS Laparoscopic - One		Robotic - Six days – Laparoscopic - Six days

Studies That Included More Than One Type of Colorectal Surgery

Studies which looked into more than one type of colorectal surgey consisted of 1266 patients [5,20-25]. The results showed again that robotic surgery had longer operative times than laparoscopic surgery [5,20-25]. Robotic surgery had less blood loss and earlier postoperative flatus and bowel opening [5,20-25]. Robotic surgery has better resection margins and less postoperative hospital stay [5,20-25]. Surgeons may now easily complete difficult colorectal resections thanks to minimally invasive surgery, which has altered the trajectory of surgical intervention for colorectal illness [5]. The robot requires some getting used to, but tests have shown that learning curves are better with robotic surgery [5]. Table 3 summarizes our results.

**Table 3:** Studies That Included More Than One Type of Colorectal Surgery SIRC = single incision robotic colectomy; SILC = single inclsion laparoscopic colectomy; mins = minutes; ml = millilitre; cm = centimetre

Source	No of Patients	Operative Time	Blood Loss	Post Op flatus and bowel opening	Conversion rate	Anastomosis leak	Wound Infection	Any dysfunction	Other Complications	Resection Margin	Hospital stay
T C Chang et al [20]	Single Incision Robotic colectomy (SIRC) (seven right hemicolectomy, one left hemicolectomy and one anterior resection) 136 Single Incision Laparoscopic Colectomy (SILC) (65 right hemicolectomy, nine left hemicolectomy and 62 anterior resection)	SIRC - 185 ± 46mins VS SILC - 208 ± 53 mins	SIRC -12 ± 22 ml VS SILC - 85 ± 234 ml f	-	SIRC - Zero VS SILC - One	-	-	-	SIRC - (One ileus) VS SILC – Three ( Two ileus) (one pneumonia)	Distal margin SIRC 6.5 ± 6.1cm VS SILC - 9.2 ± 6.7cm	SIRC - 8.3 ± 1.7 days SILC - 9.3 ± 6.5 days
Sergio Heurta et al [21]	Robotic - 105 patients Laparoscopic - 168 patients	Robotic - 276.0 ± 111.5 mins VS Laparoscopic - 171.7 ± 54.9 mins	Robotic - 86.5 ± 114.3ml VS Laparoscopic - 93.1 ± 116.1ml		Robotic - Nine VS Laparoscopic - 22	-	-	-	-	-	Robotic - 7.1 ± 7.5 days Laparoscopic - 6.6 ± 5.0 days
Elizabeth McCarthy et al [22]	Robotic - 48 patients Laparoscopic - 51 patients		Robotic surgery had less blood loss than laparoscopic and open						No difference in morbidity or mortality.		Robotic surgery had a lower length of stay
Xiao-Long Zhu et al [23]	284 patients Robotic - 104 Laparoscopic - 180	Robotic - 201.1 mins VS Laparoscopic - 195.4 mins	Robotic - 166.88 ± 106.40 ml VS Laparoscopic -- 125.08 ± 113.34 ml	robotic – 3.4 days VS Laparoscopic - six days						Lymph nodes harvested: Robotic – 12 VS Laparoscopic -12.6	Robotic – 10.7 days Laparscopic - 14.3 days
Salah Abdel Jalil et al [5]	Robotic – 306 patients Laparsocopic-1783 patients				No significant differences	Robotic - Four (1.31%) VS Laparoscopic - 19 (1.07%)	Robotic Two = (0.65%) VS Laparoscopic = 14 (0.77%)		Incisional Hernia robotic - one (0.33%) VS Laparoscopic-three (0.17%) Post op pain medication Robotic - 240 (78%) VS Laparoscopic - 1444(81%)		Median - Robotic - three days VS Laparoscopic - four days
Vanitha Vasudevan et al [24]	96 patients had robotic 131 patients had laparoscopic	Robotic – 109 mins Laparoscopic – 113 mins	Less estimated blood loss in Robotic compared to Laparoscopic.		Robotic – 13 (13.5%) VS Laparoscopic -12( 9.1%)				Major Robotic – seven VS Laparoscopic - four Minor Robotic – 10 VS Laparoscopic - seven		Robotic -5.7 days Laparoscopic - 6.6 days
Bradley R Davis et al [25]	533 patients had robotic surgery and 533 had laparoscopic	Mean operative times were longer for robotic than laparoscopic (4.37 hours vs 3.34 hours respectively)			-		1.88- Robot During or after procedure VS 1.88- Laparoscopic during or procedure		11.63% - Robot during or after complications 13.70% during or after complications	-	5.74 days for Robotic VS 6.09 days for Laparoscopic

Patients who underwent robotic surgery for colorectal surgery in general found that they passed flatus quicker/sooner than laparoscopic patients [9,14,15,23]. A case-controlled study by Morpurgo et al. demonstrated that the robotic group had an early recovery of bowel function (3 ± 1 days in the robotic group (RG) compared with 4.0 ± 1.2 days in the laparoscopic group (LG)) and lower anastomotic leak rate (RG: 0 vs LG 4) but had much longer surgery time (RG, 266±41 minutes; LG, 223±51 minutes) [26]. Another study also found similar results in that robotic surgery demonstrated a more rapid return of sexual function after surgery [27]. It also reported that robotic surgery also produced less pain, faster recovery, and decreased postoperative complications as well as better outcomes in certain cases [27]. Various studies did however conclude that the cost of robotic colorectal surgery is a limiting factor [5,23,24]. Guerriri et al.'s study states that since the introduction of robotic surgery, it has proved to be safe and effective especially for complex cases in colorectal surgery [13].

Discussion

Spinoglio et al. noted that whilst robotic laparoscopic colon surgery was feasible, even at early stages prior to expected technological improvements, operating times were found by them to be longer than in standard laparoscopic surgery [28]. Yet it was noted that robotic assistance was particularly helpful in cases involving complex and precise dissections in a confined space [10]. Guerriri et al. state that since the introduction of robotic surgery, it has proved to be safe and effective especially for complex cases in colorectal surgery [13].

In assessing the potential visual advantages, the results of a study showed that robotic techniques allow the surgeon to see the images in 3D, there is more freedom and an increase in angle and degrees in movement [27]. There is a 7-degree freedom and micro-anastomosis is possible and has an ergonomic position. It has an ergonomic system which makes it easier for the surgeon to execute complex surgeries [28]. They also reduce or eradicate tremor and human error. This improves outcomes of surgery [27]. The learning curve is similar to laparoscopic training [28]. There seem to not be many barriers and it might even be easier to learn robotic surgery than laparoscopic surgery [27]. Robotic surgery provides a qualitative leap in surgical tools and the ability to provide our patients with a minimally invasive approach even when the procedures to be done are technically challenging or in "uncomfortable" anatomical regions for the average surgeon [29]. Robotic surgery does not make the treatment straightforward or easy, but it helps shorten the learning curves of less experienced surgeons by making more minimally invasive surgery possible in more patients [29].

When compared to open, laparoscopic, or transanal technique, robotic approach appears to be the best choice for treating rectal cancer because it delivers the best mix of oncological, functional, and patient recovery results [29,30]. A shift in the paradigm of surgical training is also made feasible by the digital interface of surgical robots, which shortens learning curves to make them more thorough and most importantly, lowers the morbidity and mortality linked to them [29,31]. Surgical societies should take the initiative in this transformation and set up effective training programmes for colorectal robotic surgery [29,31].

The expense of using the robotic platform is one of the major complaints [9]. The da Vinci system is expensive up front and comes with significant consumable expenditures when utilised in robotic surgery [9]. The initial instrument costs of the robotic and laparoscopic platforms were not taken into account in a recent US national database [9]. They have included all colonic resections and furthermore there is no stratification of the type of anastomosis performed or whether any cases included intra-corporeal anastomosis [9]. Additionally, they left out the operating time of the surgery, which can serve as a stand-in for the surgeon's learning curve [9]. Numerous studies have shown that surgeons' learning curves also have an effect on expenses [9]. According to research by Morelli et al., the last stage of the learning curve was related with a considerable reduction in operating time and lower operating room expenses [32].

Muller shows that the Miskovic complexity score correlates with the rate of major complications in robotic colorectal surgery, especially if the surgeon is not proficient in robotic surgery [33]. Therefore, the learning curve should be used and taken as an important factor, when selecting complex cases to be perfomed via robotic approach [33]. This will lead to a significant reduction in major complications over time [33].

Limitations

The main limitations of this study are that these are retrospective analyses of case studies, with few comparative studies being propensity score match. Control case studies should also be carried out to prove and enhance our results further. More clinical trials and metanalysis are needed to assess the long-term results. More trials on more patients are needed to assess the benefits of robotics. Some studies were also limited to few patients and the abilities of certain surgeons and therefore more trials on more patients are needed to assess the benefits of robotics in colorectal surgery.

## Conclusions

Robotic surgery has more positives that outweigh the drawbacks and can be useful and helpful in colorectal surgeries. However further advancements are needed to minimize costs and perioperative times, due to prolonged time for setup. Implementation and training for use will also need improvement. Robotic surgery helps due to the 3D view and free movement as well as reduction in tremor. The precision is also high and loss of blood is decreased as well as a reduction in incision size. Robotic surgery has similar outcomes for right hemicolectomy but has better outcomes, less blood loss and lower conversion rate for anterior resection. However as explained the costs are high and the time to set up increases perioperative time. Overall, the future of robotics and uses in colorectal surgeries are very promising.
